# Biometric recognition of newborns and young children for vaccinations and health care: a non-randomized prospective clinical trial

**DOI:** 10.1038/s41598-022-25986-6

**Published:** 2022-12-29

**Authors:** Tom Kalisky, Steven Saggese, Yunting Zhao, Daniel Johnson, Maya Azarova, Lilia Edith Duarte-Vera, Lucila Alejandra Almada-Salazar, Daniel Perales-Gonzalez, Enrique Chacon-Cruz, Jiaxing Wang, Rishi Graham, Alexandra Hubenko, Drew A. Hall, Eliah Aronoff-Spencer

**Affiliations:** 1 University of California San Diego, La Jolla, CA USA; 2 Universidad Autonóma de Baja California, Campus ECISALUD, Tijuana, Baja California Mexico; 3 Hospital Central Tijuana, Tijuana, Baja-California Mexico; 4 University of California San Diego School of Medicine, La Jolla, CA USA

**Keywords:** Population screening, Biomedical engineering, Optoelectronic devices and components

## Abstract

Although universal biometrics have been broadly called for, and there are many validated technologies to recognize adults, these technologies have been ineffective in newborns and young children. The present work describes the development and clinical testing of a fingerprint capture system for longitudinal biometric recognition of newborns and young children to support vaccination and clinical follow-up. The reader consists of a high-resolution monochromatic imaging system with an ergonomic industrial design to comfortably support and align infant fingers for imaging without a platen. This imaging approach without a platen, also called free-space imaging, reduces fingerprint distortion and ensures a more consistent finger placement. This system was tested in a newborn ward and immunization clinic at an urban hospital in Baja, California, Mexico, from 2017 to 2019. Nearly five hundred children were enrolled and followed for up to 24 months. With a protocol of imaging all ten fingers, the failure to enroll (FTE) rate was < 1% when acquiring at least two fingers for all ages and < 2% when enrolling at least four fingers. The verification (1:1) true accept rate (TAR) was 77% for newborns enrolled at ≤ 3 days of age and 96% for those enrolled at ≥ 4 days of age, both at a time gap of 15–30 days after enrollment at a false accept rate (FAR) of 0.1%. Using the top-ranked match score, the identification rate (1:many) was 86% for the ≤ 3 days enrollment age and 97% for age ≥ 4 days for a single finger at 15–30 days after enrollment. The enrollment protocol and the frequency of updating will increase for infants compared to adults. However, these data suggest that a high-resolution, free space imaging technique may fill the final gap for universal biometrics across all populations called for by the United Nations Sustainable Development Goal 16.9.

## Introduction

The United Nations Sustainable Development Goal 16.9 calls for the legal identity of all people worldwide, including birth registration, by 2030^1^. Today, over 1 billion people worldwide still lack this legal identity, and nearly one-half of these are children^2^. A growing number of countries have closed this gap by implementing national identity programs that use biometrics such as face, eye, and fingerprint scanning to create digital ID systems^3^. The largest of these, Aadhaar, has been in place in India since 2019 and has enrolled over 1.1 billion citizens^4^. This program has also produced some controversy^5,6^, and among its most notable gaps has been the exclusion of children under five, as adult biometric technologies have failed to meet standards for legal use^7^.

Biometric recognition is most commonly associated with a criminal investigation, access control, and verification for financial services^8^. While these applications have focused on adult populations and are the primary uses of biometrics today, many needs remain unmet as existing biometric technologies have failed for children under five, particularly under one year of age. A study of age and aging in fingerprints revealed that the most challenging age group was 0–4 years^9^. Fingerprint analysis for this group suffered from very poor fingerprint image quality, poor accuracy using standard devices, and a pronounced aging effect^10–13^.

Biometrics are increasingly integrated into development and health programs to assure beneficiary identity and verify that goods and services reach their targets. A systematic review of over 160 biometric programs reports that biometrics has consistently “improved treatment and program administration"^14^. A 2015 study from Uganda, comparing direct observed therapy (DOT) based tuberculosis treatment with and without a biometric-linked health record, reported higher cure (55.6% versus 28.3% [*P* < 0.01]) and lower loss to follow up (0% versus 7%) in the intervention arm^15^. Similar results were obtained in a study in Malawi by Brown University School of Public Health that found a biometric system captured nearly 50% of HIV visits missed by the current EMR system^16,17^.

Guidelines for biometric use in newborns and children vary widely worldwide, with some countries passing laws mandating biometric registration of births and deaths (Brazil, Bangladesh). Today, many biometric methods available for adults meet the standard for National ID programs. These include iris and retinal eye scanning, facial recognition, and finger and palm imaging^18^. These modalities have been tested on children with commercially available devices, and each has shown significant failure modes in real-world settings^10,12,19,20^. In the cases of the three most widely accepted and used modalities (eyes, face, and finger scanning), each has a unique barrier to reliable recognition in children under 1 year of age (Table S1).

Emerging technologies have shown promise to lower the age of accurate biometric recognition. These include work by Engelsma et al*.*^11^, who have significantly reduced the enrollment age and recognition accuracy using high-resolution contact fingerprint scanners, identifying children as early as three months (reported true accept rate,TAR (at a false accept rate (FAR) of 0.1%) of 64.7% for an enrollment age of 0–1 months, and TAR (FAR 0.1%) 92.8% for enrollment ages between 2–3 months with three months between enrollment and authentication). Weingaertner et al*.*^21^ have likewise shown promising results using high-resolution palm printing, and Tiwari et al*.*^19^ have studied novel modalities such as ear recognition. However, none of these approached the standard set by Engelsma et al*.*^11^. While each group has made gains in lowering the age of accurate enrollment for children, the lower bound remains greater than three months and, more realistically, one year in most contexts. In the case of finger and palm prints using successively higher resolution imagers by contact-based methods, the lower bound of fingerprints seems to be set by physiologic changes in the infant that allows their more pliable skin to deform upon contacting the scanner. Likewise, face and ear scanning has met challenges as infant facial and ear structure ages, and infant compliance and caregiver acceptance cases have challenged use in clinical settings.

As a result, even with advances by Engelsma and others, the best-reported technologies still fall short of the targeted performance for newborns and infants, leaving a vital gap in the identity chain. This work discusses the results of a prospective biometric trial at an urban medical center in Baja, California, Mexico, implementing a newly developed contactless fingerprint technology to close this gap.

## Methods

### Trial methods, location, and context

We recruited 494 newborn and young infants at a public hospital in Tijuana, Mexico, between January 1, 2018, and September 12, 2019. The public hospital is the primary care facility for the community where vaccinations are administered, thus providing a high volume of subjects who would return to the study location for follow-up. Informed parental consent to participate as well as informed parental consent to publish identifying information and images was obtained, and all work was performed following the relevant guidelines and regulations with IRB approval from UC San Diego (IRB #151400) and ISRCTN clinical trial registration (ID ISRCTN14852287, 24/3/2022). Enrollment occurred in the newborn ward and vaccine clinics, with follow-up at the vaccine clinic in the hospital. Parents and caregivers were conveniently recruited during daytime business hours through a simple Spanish language handout explaining the study. Subjects were included into the study upon parental acceptance and received a small monetary compensation for each visit.

### Biometric technology

**Figure 1:**
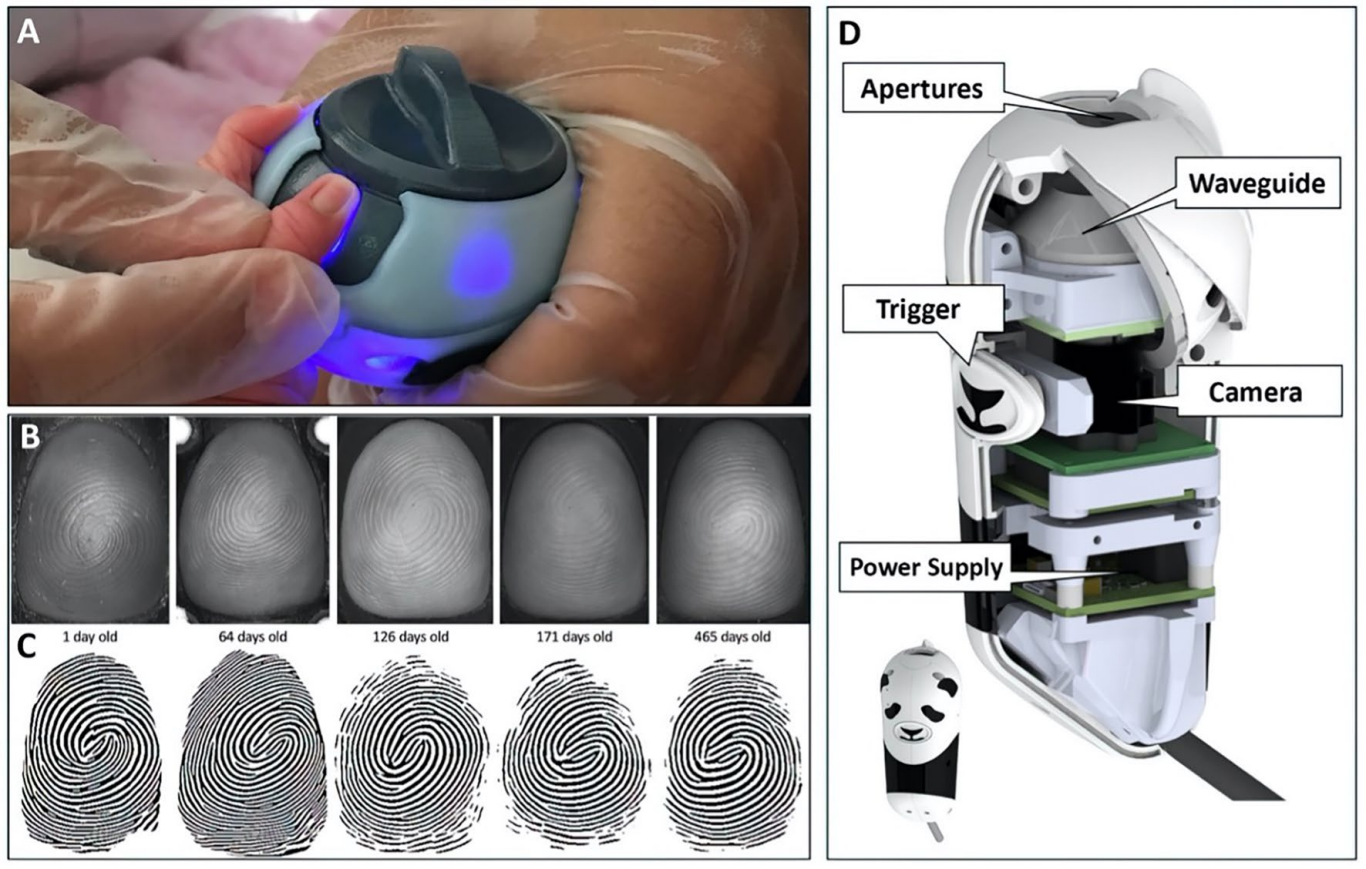
The biometric device and example imagery used in this study. (**A**) the device in use, (**B**) time-course images of a single subject’s finger, (**C**) binarized prints finger images, and (d) the internal components and design of the “Panda” device.

We employed a human-centered design process to reframe the identification problem and iterate solutions with beneficiaries as previously described^22,23^. Based on observed ergonomic challenges interacting with infants and contact-induced deformation of fingerprints with traditional scanners, we developed a platen-free optical approach that supports an infant finger with appropriately sized apertures while allowing the acquisition of high-resolution images of fingerprint minutiae without disrupting the finger surface. Figure 1A shows the device in use. The dial allows the operator to rotate in apertures of various sizes to support fingers of different ages.

Figure 1B shows a series of images of a single subject’s finger from 1 to 465 days of age captured with the 5-megapixel (MP) monochrome CMOS sensor with internal blue LED illumination. The image processing pipeline previously described in^23^ was used to improve the image contrast and normalize the ridge-to-ridge distance to account for the infant's growth over time. Figure 1C shows the binarized print determined using the VeriFinger SDK for each corresponding finger image of a single individual over 465 days. Figure 1D shows the internal design of the device, nicknamed “Panda.”

### Performance evaluation

We employed standard models of detection error trade-off (DET) to determine verification (1:1) performance and Comprehensive Match Characteristics (CMC) analysis for identification (1:many). Failure to enroll (FTE) rates were determined on a finger-by-finger basis using a predetermined fingerprint quality threshold. Multi-finger biometric fusion was carried out using the average matching scores of multiple fingers, as reported by Jain et al*.*^24^.

## Results

### Cohort characteristics

We recruited 494 children ages 0–329 days old at first enrollment (min 0, max 329, avg 32) with longitudinal follow-up for up to 19 months for some subjects (Table 1). Since newborns usually stay in the hospital for 1–3 days, we divided the subjects into two separate age groups; 1–3 days and 4 days and up. We enrolled 297 newborns (age = 3 days) and 197 newborns (age = 4 days). There were 253 subjects that had at least 1 additional visit (min 1, max 7, avg 2.4) for a total of 1166 scan sessions and 1704 paired visits with an average time between visits of 33 days (min 6, max 576, avg 33). There were 241 subjects that did not return to the hospital for vaccinations, thus they were only enrolled and did not have any subsequent scans for inclusion into the verification and identification performance analysis.

**Table 1:** Cohort characteristics.

**Demographics**	
# Of subjects	494
Females/males	236/258
% F/% M	48% / 52%
**Age at first enrollment**	
0–3 Days	297
> 4 Days	197
**Scan sessions**	
Only enrolled	241
1 Follow-up visit	71
2 Follow-up visits	52
3 + Follow-up visits	132
# Of total visits	1166
Average time between scans	33 d
# Of paired visits	1704

### Effect of enrollment age on failure to enroll

We first investigated the effect of age on fingerprint quality and successful enrollment. Using the Neurotechnology VeriFinger fingerprint identification software development kit (SDK), the average image quality of infants enrolled from ≤ 3 days was 49.3, and for infants enrolled at ≥ 4 days was 60.2, which is indicative of the fingerprint issues of newborns^23^. As a result of these quality differences, the failure to enroll (FTE) rates varied slightly by enrollment age. Figure S1 shows the FTE rate for both enrollment ages for all minimum number of fingers, using a VeriFinger template quality score threshold of 40. Using this quality threshold, we enrolled at least 2 fingers from any subject > 99% of the time, taking a minimum of 5 images from any individual finger. The FTE rate steadily increases for all children to ~ 2% when enrolling a minimum of 5 fingers and up to ~ 20% for all 10 fingers for the earlier enrollment age. Since the protocol for this study was to attempt scans on all fingers, the specific fingers used for each subject varied.

### Effect of age on verification performance

**Figure 2:**
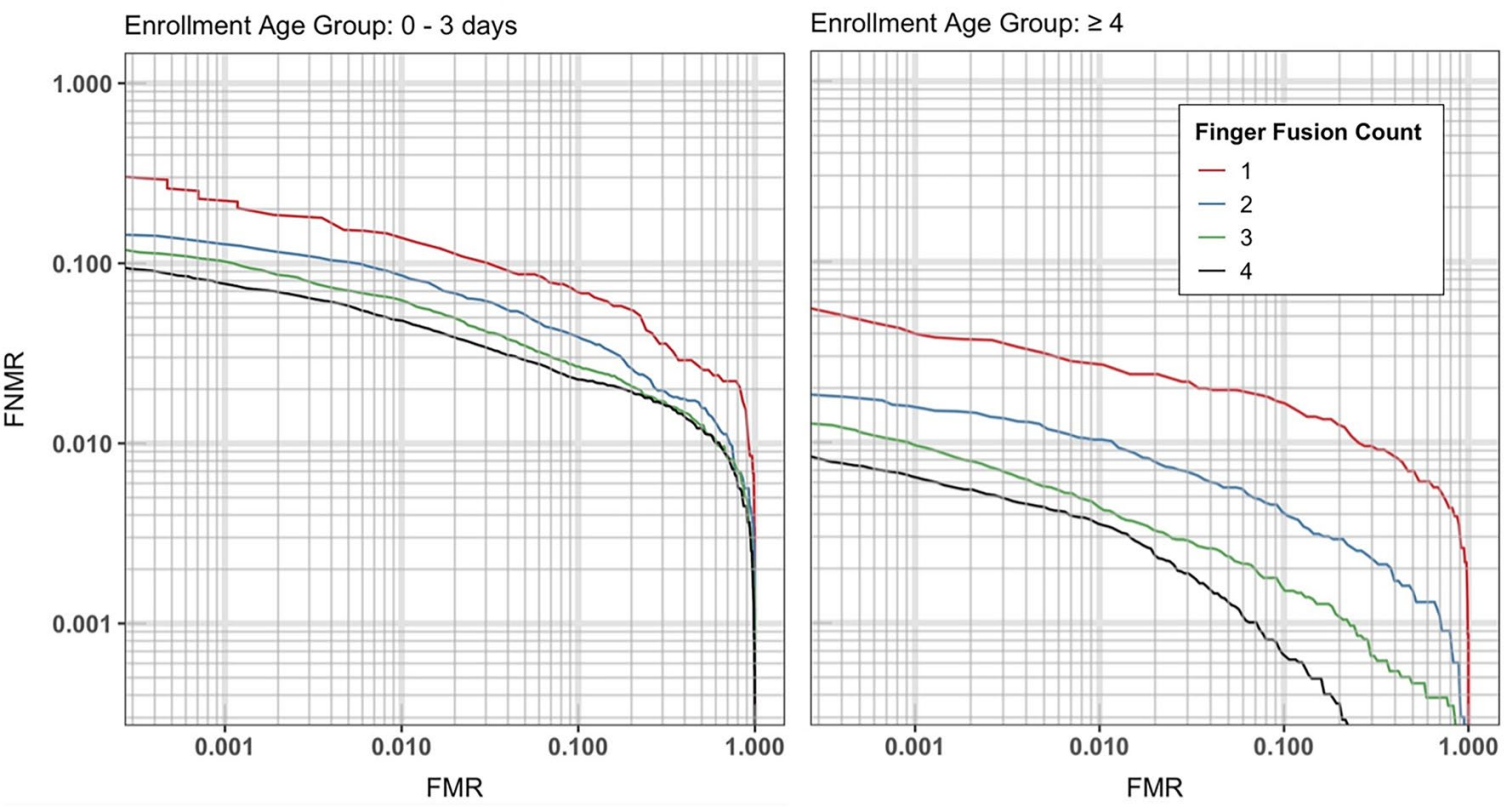
Effect of enrollment age and number of fingers used on the verification performance for (Left) 0–3 day age enrollment and (Right) > 3 day age enrollment with a verification time gap of 15–30 days.

We evaluated using verification performance using single and multiple fingers (*i.e.,* finger fusion). For finger fusion, the average match score for multiple fingers for a subject was used for the threshold for verification. Figure 2 shows the DET curves for ≤ 3-day age enrollment (left) and ≥ 4-day age enrollment (right) for various finger fusion counts. For these data, the days between enrollment and verification were ≥ 15 and ≤ 30 days. The performance improves for the later enrollment and when multiple fingers are used. For early age enrollment, the single finger False Non-Match Rate (FNMR) at a False Match Rate (FMR) of 0.1% was 23%, and for the later enrollment age, the FNMR was reduced to 4.0%. Fusion of multiple fingers significantly improved performance for both age groups, with a 4-finger fusion yielding an FNMR of 7.7% for the lower age enrollment group and 0.63% for the older enrollment group. Table 2 summarizes the TAR for the two enrollment ages and different finger fusions at the 0.1% FMR.

**Table 2:** TAR for the two enrollment ages and different finger fusions at the 0.1% FMR for 1, 2, 4, and 10 finger-fusion.

True accept rate @ FMR = 0.1%		
# of Fingers fused	Age at enrollment	%TAR
1 finger	0–3 days	0.77
	≥ 4 days	0.96
2-fingers	0–3 days	0.87
	≥ 4 days	0.98
4-fingers	0–3 days	0.92
	≥ 4 days	0.99
10-fingers	0–3 days	0.97
	≥ 4 days	1

**Figure 3:**
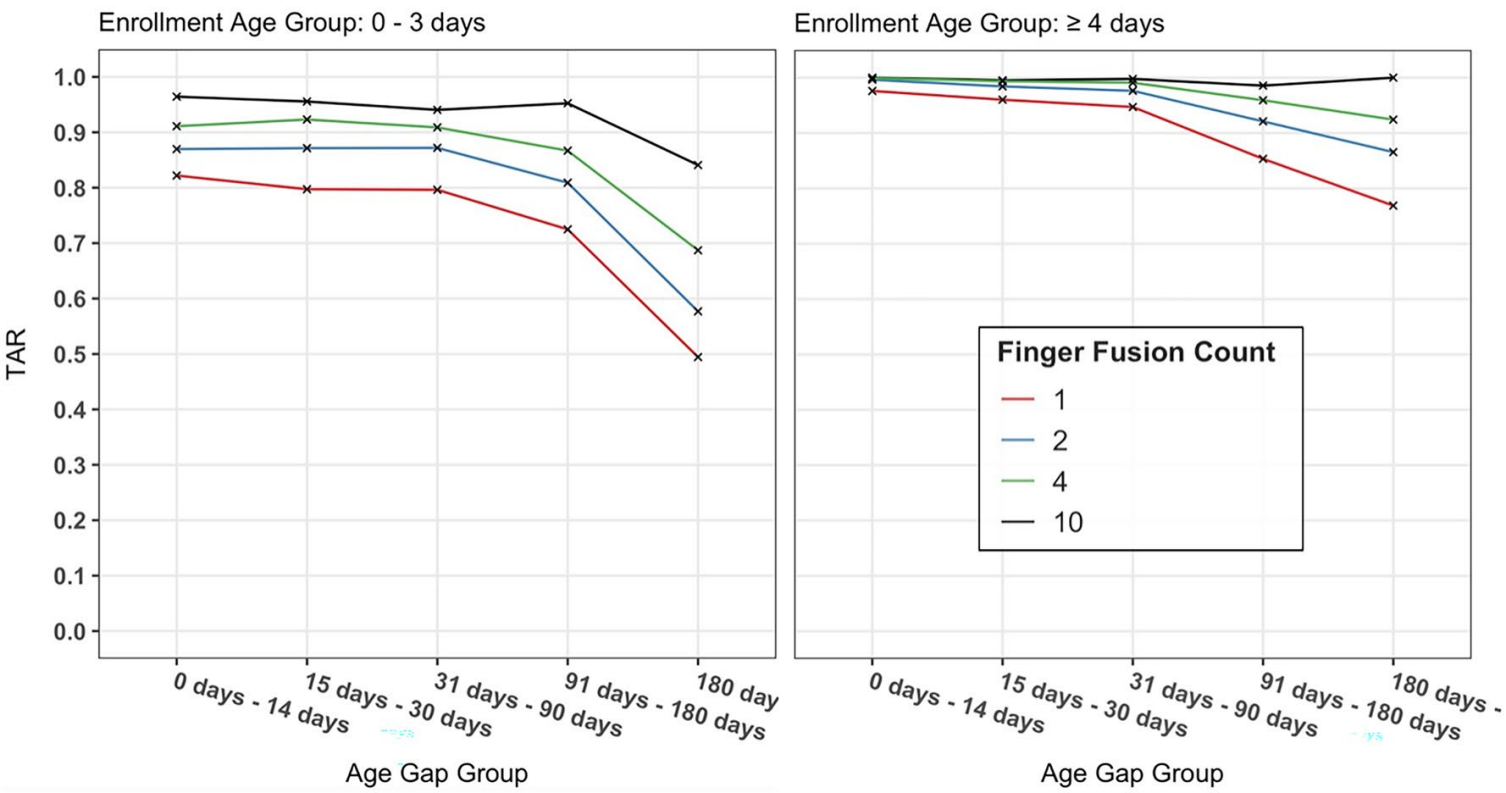
The effect of age at enrollment and time gaps between enrollment and verification up to 180 days, for (Left) enrollment age group 0–3 days and (Right) for the enrollment age group of 4 days and older.

The time interval between visits also affected the verification performance, which was similarly dependent on age at enrollment. Figure 3 shows that the TAR (all @ FPR 0.1%) improves dramatically for all finger fusion numbers when the enrollment age increases from ≤ 3 days (left) to ≥ 4 days (right). As the age gap between enrollment and verification increases, the performance decreases only slightly up to the 31 to 90 days gap, with an increased performance reduction in the > 90 and > 180-day time gaps. For a single finger enrollment at birth (≤ 3 days), the TAR is around 80% up to 90 days and reduces to 50% at greater than 6 months. The 4-finger enrollment for that age improves to 90% and 70% for the same age gaps, respectively. For the later enrollment age of ≥ 4 days, the TAR stays well above 90% for all fingers up to the 90-day gap range, with a reduction in TAR to 77% for a single finger > 6 months, above 90% for 4 fingers, and 100% for 10 fingers.

### Identification performance

**Figure 4:**
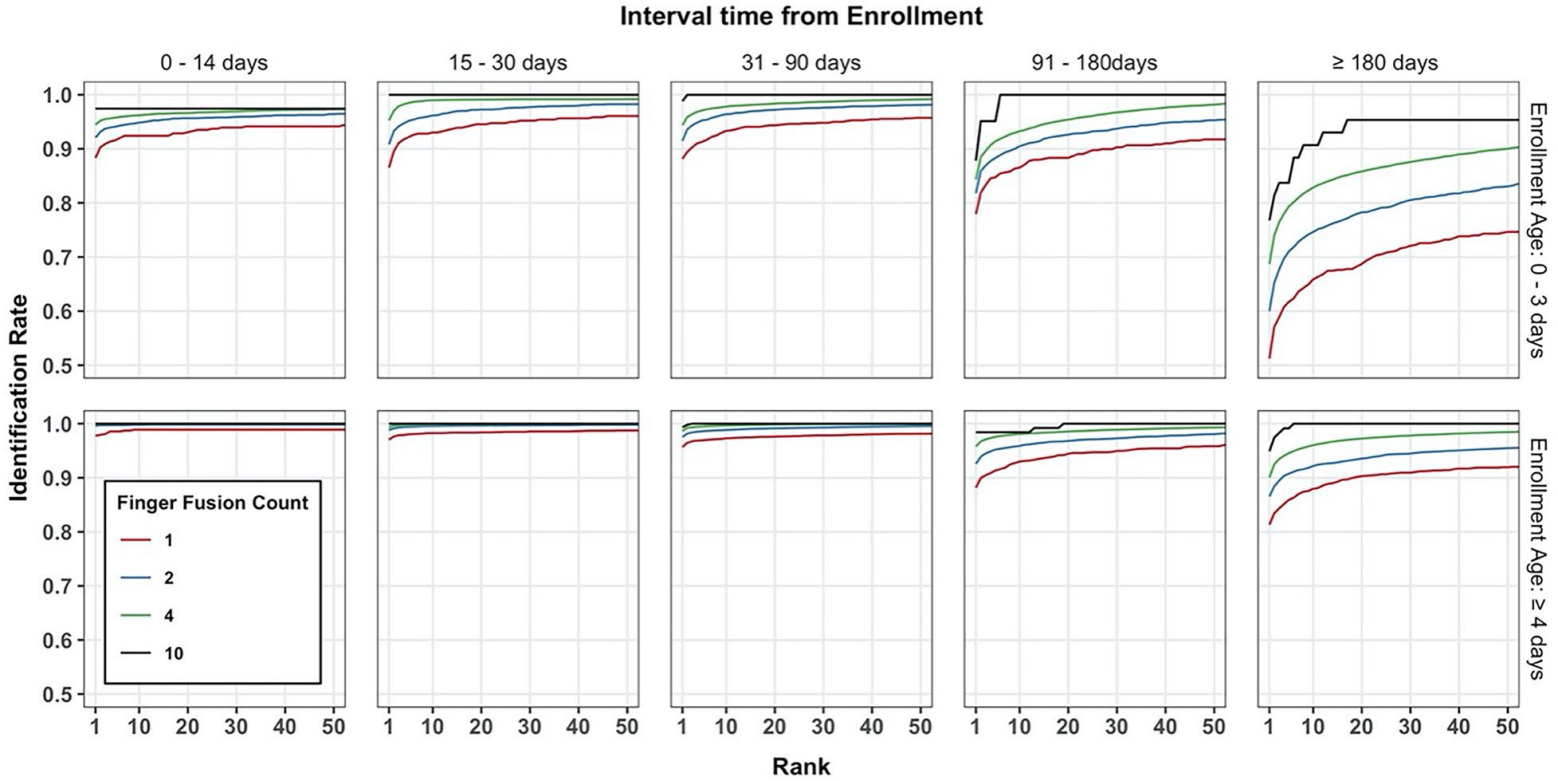
CMC analysis as a function of enrollment age, time gap, and the number of fingers.

Finally, we assessed the comprehensive match characteristic (CMC) to determine the ability to identify a single individual amongst many. The CMC analysis as a function of enrollment age, time gap, and the number of fingers used are shown in Fig. 4. Much like the verification data, we saw improvement at the later enrollment ages and smaller time gaps.

## Discussion

Here we report a comprehensive analysis of the first clinical study to utilize platen-free imaging for the biometric recognition of newborns and infants. The platen-free imaging overcomes issues of deformation introduced by current methods but adds new complexity to the interaction between children and biometricians and the manufacture of the technology^23^. We found that the attention to ergonomic design and participation of caregivers in the design process facilitated both clinical workflow and the willingness of participants to join and continue in the study. Preliminary qualitative usability studies were positive but require further investigation^22,23^. Time-to-enroll and verification were difficult to quantify in the study due to frequent and necessary interruptions; however, enrollment could generally be accomplished in under five minutes, with verification times between 20 s and 2 min.

Our quantitative analysis demonstrated a significant improvement in the recognition rate over currently reported modalities, yielding accuracies that might now be considered acceptable for clinical applications; single finger TAR (FAR 0.1%) of 77% for 0–3 days and 96% > 3 days. For comparison, Engelsma et al*.* reported TAR (FAR 0.1%) of 64.7% for an enrollment age of 0–1 months at an interval of 3 months^11^. We found that quality strongly trends with age in the first days of life, and notable failure modes continue to include skin sloughing and the extremely fine newborn features, which are most pronounced in the first 72 h after birth but can persist for 6 and even 12 weeks. Early enrollment or long intervals between visits both showed decrements in accuracy. Due ot the limitations of the duration of this study, the need for re-enrollment as the child ages was not conclusive. Thus it might be beneficial, where possible, to periodically re-enroll children to minimize the time interval for verification. For the case of those first enrolled at birth, periodic re-enrollment might be critical to ensure the highest accuracy for legal identity, whereas less frequent data collection may suffice if children are enrolled weeks or more after birth.

This study had other limitations. We deployed a prototype scanner with our intervention conducted in parallel to existing critical clinical workflows, with the characterization of performances conducted post-hoc. This analysis limited the time to enroll, verify and identify, or perform workflow and algorithmic optimizations. We also had little data in the 24–48-h enrollment period (> 95 of newborns were enrolled within 18 h of birth), nor did we collect significant data on verification at 2 or 3 days. The majority of revisits occurred at 2 weeks or greater. Finally, the trial scale is still limited in the diversity and size of the population compared to those expected by the National Institute for Standards and Technology^25^ and other biometric certifying organizations.

It is evident from this effort that special biometric hardware and fingerprint collection protocols will be needed for newborn applications. Unlike adults, where the collection of images from only several fingers may be sufficient, these data suggested that as many fingers as possible should be collected for enrollment for newborns. If possible, the initial enrollment should occur after at least 4 days of age to optimize performance. Re-enrollment on subsequent visits should also be conducted to minimize the time gap between scans and limit any issues associated with a child’s physiological changes during growth that are not adequately corrected with the size normalization image processing used for this study.

## Conclusion

We demonstrate the feasibility of using a free space fingerprinting technique to improve verification and identification for newborns and young children in the hospital during immunizations or ambulatory care. This technique closes a vital gap in the biometric toolkit and presents a step toward the UNDG 16.9 calling for the identity for all. Our study found significant effects of age and aging on all aspects of the biometric encounter, requiring more investigation and technology refinement and directing one to the technology's strengths and limitations. For instance, verification of any age group over short periods, as might be expected during immunizations, enrollment, and verification of 3–4 fingers would yield > 95% TAR, which would hold for months and perhaps years for non-newborns. At the same time, up to 10 fingers would be required to reliably identify an unknown individual many years after birth, a problem that could be solved with periodic re-enrollment during routine childhood care or require other factors to assure accurate identity if no intercurrent enrollment occurred. While we did not directly measure the effect of our technology on the delivery of vaccines, our time to identify and enroll was notably similar to that observed for usual care. The qualitative usability of the tool and quantitative results of this trial support the growing body of evidence that biometrics can serve to improve the delivery of vital health care and vaccines for routine immunization or during global pandemics, enabling more effective and equitable benefits to those most in need.

## Supplementary Information

Supplementary Information.

## Data Availability

The datasets generated and analyzed during the current study are not publicly available because the data contains biometric data of participants and cannot be published but are available from the corresponding author on reasonable request.
