# Supplementary material for: Biometric recognition of newborns and young children for vaccinations and health care: a non-randomized prospective clinical trial
Source: Sci Rep. 2022 Dec 29;12:22520. doi: 10.1038/s41598-022-25986-6 (PMC9800356; doi:10.1038/s41598-022-25986-6)
Supplement: Supplementary file 1 — Supplementary Information. [file 41598_2022_25986_MOESM1_ESM.pdf]

Biometric recognition of newborns and young children for vaccinations and health care: A non-randomized prospective clinical trial

[1]Tom Kalisky^, [1]Steven Saggese^, [1]Yunting Zhao^, [1]Daniel Johnson, [1]Maya Azarova, [2]Lilia Edith Duarte-Vera, [2]Lucila Alejandra Almada-Salazar, [2]Daniel Perales-Gonzalez, [2,3]Enrique Chacon-Cruz, [1]Jiaxing Wang, [1]Rishi Graham, [1]Alexandra Hubenko, [1]Drew A. Hall, [1,4]Eliah Aronoff-Spencer*

[1]University of California San Diego, La Jolla, California, USA

[2]Campus ECISALUD, Universidad Autonóma de Baja California, Tijuana, Baja California, Mexico

[3]Hospital Central Tijuana, Baja-California, Mexico

[4]University of California San Diego School of Medicine, La Jolla, California, USA

^Authors contributed equally

***Corresponding Author:**

Eliah Aronoff-Spencer, MD PhD

Earonoffspencer@health.ucsd.edu

Supplementary Figure S1: *Failure to Enroll Rate (FTE) for different enrollment ages and minimum number of fingers enrolled using a quality score of 40 as the enrollment threshold score*

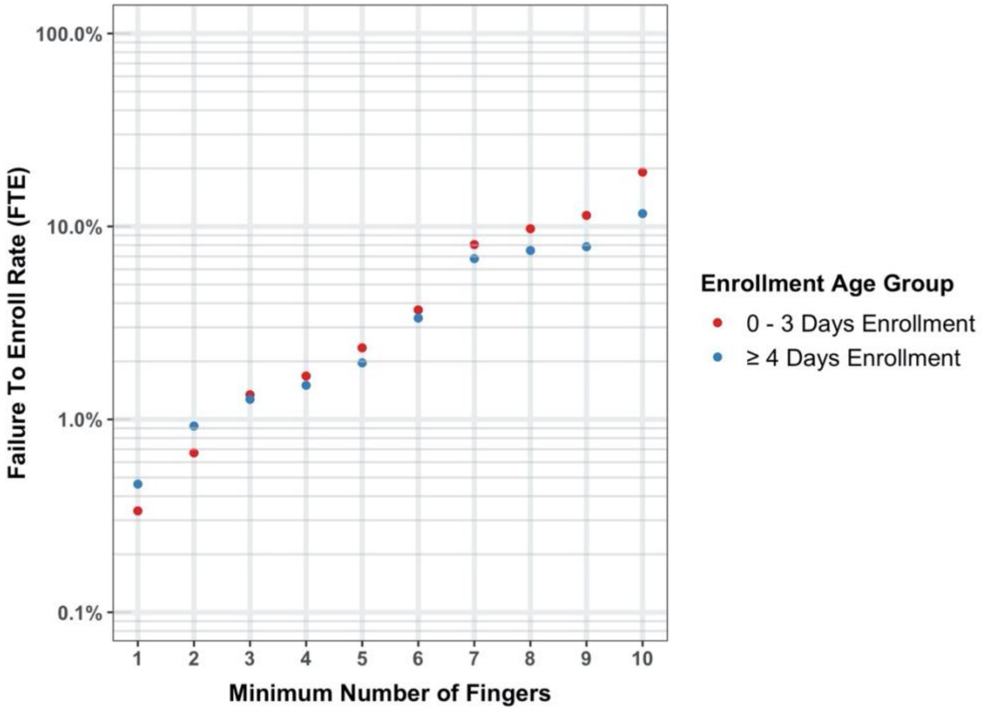

Supplementary Table S1: *Comparison of different biometric traits and their compatibility for infant identification based on information from [19,23] and the authors' perception.*

Biometric Trait	Required Degree of Subject Cooperation	Persistence	Parental Concerns	Primary Failure Modes	Examples for Attempt with Infants
**Face**	Moderate (Stare towards camera with neutral expression)	Low (Facial Aging)	Minor	Facial aging, trauma	Singh et al. (20)
**Fingerprint**	High (Allow the operator to hold the child's finger)	Potentially high	Moderate	Sloughing, worn prints	Francis Galton et al. (26), Jain et al. (27)
**Iris**	High (Open eyes and stare towards camera)	Potentially high	Major	Eye condition, behavior	Corby et al. (28)
**Palm-print**	Moderate (Open fist and allow operator to hold the palm)	Potentially high	Moderate	Sloughing, worn prints	Weingaertner, D et al. (21)
**Footprint**	Moderate (Removal of shoes and allow operator to hold foot)	Unknown	Minor	Sloughing, worn prints	Weingaertner, D et al. (21)
**Ear**	Low (Keep head steady)	Potentially high	Minor	Aging, specificity	Fields, C et al. (29)
**Palm Vein Pattern**	Moderate (Open fist and allow the operator to hold the palm)	Potentially high	Moderate	Thin, obscured vasculature in infants, fat, hair in adults	Cao, J et al. (30)
